# The “Gateway” hypothesis: evaluation of evidence and alternative explanations

**DOI:** 10.1186/s12954-024-01034-6

**Published:** 2024-06-07

**Authors:** Arielle Selya

**Affiliations:** Pinney Associates, Inc, 201 North Craig Street, Suite 320, Pittsburgh, PA 15213 USA

**Keywords:** Common liability, Diversion hypothesis, Electronic nicotine delivery systems (ENDS), Gateway hypothesis, Cigarettes, Youth

## Abstract

**Background:**

Electronic nicotine delivery systems (ENDS) offer a substantial harm reduction opportunity for adults who smoke and are unlikely to quit. However, a major concern about ENDS is their use by non-smoking youth, and particularly whether ENDS are acting as a “gateway” that leads youth to later start smoking cigarettes. However, evidence for the gateway hypothesis can be interpreted in alternative ways, e.g. that youth who have certain characteristics were already predisposed to use both ENDS and cigarettes (“common liability” explanation).

**Aims:**

This commentary provides an evaluation of the gateway hypothesis that is accessible by a lay audience. This paper first reviews and evaluates the evidence interpreted as supporting the gateway hypothesis. Important alternative explanations (i.e., common liability) are discussed, as are different types of evidence (i.e., population-level trends) that can help differentiate between these competing explanations.

**Overview:**

Evidence for the gateway hypothesis is based on the finding that youth who use ENDS are more likely to also smoke cigarettes. However, this evidence suffers from an important flaw: these studies fail to fully account for some youths’ pre-existing tendency to use products containing nicotine, and inappropriately interpret the results as ENDS use *causing* some youth to smoke. Common liability studies suggest that ENDS use does not, in and of itself, directly cause youth to later smoke cigarettes, beyond their pre-existing tendency to use products containing nicotine. Population-level trends show that youth cigarette smoking declined faster after ENDS use became common, which contradicts the central prediction of the gateway hypothesis (i.e. that youth smoking would be more common following ENDS uptake, than otherwise be expected).

**Conclusion:**

Evidence offered in support of the gateway hypothesis does not establish that ENDS use *causes* youth to also smoke cigarettes. Instead, this evidence is better interpreted as resulting from a common liability to use both ENDS and cigarettes. Population-level trends are inconsistent with the gateway hypothesis, and instead are consistent with (but do not prove) ENDS *displacing* cigarettes. Policies based on misinterpreting a causal gateway effect may be ineffective at best, and risk the negative unintended consequence of increased cigarette smoking.

## Background

Electronic nicotine delivery systems (ENDS) do not involve combustion, and thus expose people who use ENDS to substantially lower levels of harmful constituents than do cigarettes [1]. As a result, ENDS offer a substantial harm reduction opportunity for adults who smoke and are unlikely to quit in the near term [2, 3]; however, ENDS’ possible beneficial effects must be weighed against their possible detrimental effects in order to understand their net impact on population health. ENDS could benefit individuals if they are used instead of cigarettes, including among people who smoke cigarettes and switch completely to ENDS (when they would have otherwise continued smoking), and people who initiate ENDS and as a result avoid cigarettes (when they would have otherwise initiated smoking). On the other hand, ENDS could pose harm if taken up by people who would not otherwise smoke, including among those would have otherwise quit smoking, and among people who would have otherwise never used products containing nicotine – especially if ENDS use causes them to also smoke cigarettes (the “gateway” hypothesis).

The latter possible pathway is the focus of this commentary, i.e. that ENDS use by non-smoking youth could act as a gateway that leads youth to later start smoking cigarettes [4, 5] – which are at the high end on the continuum of harm [6–8]. If the gateway hypothesis were true, it would reduce the likely public health benefits that ENDS provide as alternatives for adults who smoke.

The evidence provided in support of the gateway hypothesis is the finding that youth who use ENDS are also more likely to also smoke cigarettes [4, 5, 9]. The gateway hypothesis is that this association reflects a causal link: that using ENDS *causes* youth to smoke cigarettes, when they would otherwise have not.

However, this causal gateway hypothesis is not the only way to interpret these findings. It is also possible, for example, that youth who have certain characteristics were already predisposed to use both ENDS and cigarettes (and possibly to engage in other behaviors) [10–13]. In other words, what initially may *appear* to be a causal association between ENDS use and cigarette smoking, is likely instead *better* explained by other factors (e.g. home and social environment, personality characteristics, mental health or emotional challenges) predisposing some people to use products containing nicotine in general, including both ENDS and cigarettes [10–13]. In this “common liability” explanation of the observation that youth who use ENDS also smoke, youth who have these pre-existing characteristics are likely to have smoked cigarettes anyway, regardless of whether or not they used ENDS first.

Different lines of evidence can also help figure out whether the gateway hypothesis or the common liability explanation is more likely to be true. For example, looking at cigarette smoking trends across the entire youth population in the US can be a “reality check” [14] that can help to choose between these competing explanations [15, 16]. For example, if the gateway hypothesis were true, it would mean that as ENDS were introduced and came to be used by youth, one should see a rise in youth cigarette smoking, compared to what would have occurred in a world without ENDS.

The question of whether there is a causal gateway association is critical to inform effective policies for nicotine and tobacco products. Many papers that interpret the evidence in favor of a causal gateway explanation conclude by recommending policies and restrictions on ENDS in order to prevent youth use of all products containing nicotine – including cigarette smoking [4, 9, 17] – for which a causal association is logically necessary (though not sufficient) to achieve the goal of curbing cigarette smoking. However, if the association is *not* causal, such policies will not only be ineffective in achieving their goal, but also could result in harmful unintended consequences if the substitutability between the two products is not recognized. Indeed, quasi-experimental studies have found that some policies restricting ENDS have unfortunately resulted in increased *cigarette* smoking [18–20], which is objectively more harmful. Thus, accurately understanding the nature of the association between youth e-cigarette use and cigarette smoking is essential for effective policy.

This commentary intends to provide a lay-audience-accessible review of the above evidence, with respect to the gateway and common liability explanations. Specifically, this commentary:

- Provides a description of the “gateway” explanation and the supporting evidence;
- Evaluates the strengths and weaknesses of this evidence;
- Looks at other ways of interpreting the same evidence, especially the common liability explanation; and.
- Considers what other types of evidence (e.g. population trends) can tell us about the gateway vs. common liability explanations.

## The gateway hypothesis

The idea of one substance acting as a gateway to another originated from observations that youth often exhibited the following sequence of substance use: (1) wine or beer, (2) cigarettes or hard liquor, (3) marijuana, and (4) illicit drugs [21, 22]. One focus of this work was the observation that illicit drug use rarely occurred without first using marijuana, leading to speculation that marijuana was a gateway to illicit drug use [21, 22]. While it is no longer accepted that marijuana acts as a gateway to illicit drugs [23], a similar concern is currently expressed about ENDS use acting as a gateway to cigarette smoking among youth – despite the fact that the flaws in the marijuana gateway concept apply more generally to the question of earlier stages in the sequence of substance use causing later stages [23]. Under the ENDS gateway hypothesis, ENDS use is thought to *cause* some youth to later smoke; in other words, it is believed that these youth *would not have smoked cigarettes if they had not first used ENDS*.

At first glance, many studies appear to support the explanation that ENDS act as a gateway to cigarette smoking among youth. For example, a study from the Truth Longitudinal Cohort found that 27.5% of never-smoking youth and young adults who used ENDS in 2018 had gone on to smoke at least one cigarette by 2019, versus only 2.4% of those who *never* used ENDS [9]. Similarly, among students in California and Connecticut who never smoked, 15.1% of those who used ENDS later began experimenting with smoking, versus only 4.4% of those who never used ENDS [24]. Similar findings have been consistently found in many similar studies: in fact, the National Academies of Sciences, Engineering, and Medicine concluded, after reviewing the available evidence, that “there is **substantial evidence** that e-cigarette use increases risk of ever using combustible tobacco cigarettes among youth and young adults” [5].

This evidence could be interpreted as evidence that ENDS use is a gateway to cigarette smoking, because this result would be expected if the gateway explanation were true. However, this evidence does not actually show that ENDS are a gateway to cigarettes. Observing an association between two behaviors in the same persons does not demonstrate that one causes the other. The next section evaluates the strengths and weaknesses of the evidence relevant to the gateway hypothesis.

## Evaluation of the evidence for the gateway explanation

### Strengths of the evidence for the gateway explanation

There are some strengths to the above evidence for the gateway explanation that its supporters find compelling.

First, there is consistency across many different studies in the findings that youth who use ENDS are significantly more likely to later smoke cigarettes. When different studies consistently report the same finding, this is considered even stronger “higher-level” evidence. It is clear that the association between ENDS use and smoking is consistent [4, 5]; but this would also be true even if the association were not causal.

Second, many studies supporting the gateway hypothesis have the necessary time sequence, i.e. that ENDS use occurs first, followed by cigarette smoking at a later time. Some studies supporting gateway do *not* look at which product was used first, so might include youth who smoked cigarettes before using ENDS (the wrong time sequence for gateway). However, other studies that do focus on the correct time sequence (i.e. ENDS use among youth who had not (yet) smoked) show the same results: that youth who used ENDS are significantly more likely to smoke cigarettes later.

Third, there are believable explanations as to *how* a gateway effect might work. If the gateway hypothesis that ENDS use *causes* youth to smoke cigarettes is true, there should be a sensible explanation for *how* exactly it might work. Several explanations have been suggested, such as that ENDS could make youth dependent on nicotine, which could cause them to also seek out cigarettes, or that using ENDS could make cigarette smoking seem more socially acceptable or view smoking more favorably [5]. Additionally, inhaling e-liquid aerosol could acclimatize youth to obtaining nicotine via inhalation, which could make a transition to cigarettes easier versus starting from a different type of nicotine product (e.g. smokeless tobacco or nicotine pouches). These ideas about *how* a gateway effect might work are sensible, and this makes the gateway idea believable.

### Weaknesses of the evidence for the gateway explanation

Despite the apparent strengths discussed above, none of the evidence conclusively demonstrates that the causal gateway hypothesis is true. The critical flaw in the evidence for the gateway hypothesis is that, according to the common-liability explanation, youth who use ENDS already had a tendency to smoke cigarettes; thus, it may not have been the ENDS use that *caused* these youth to smoke cigarettes. In other words, even if these youth had not used ENDS, they would have probably gone on to smoke cigarettes anyway. This possibility goes against the gateway hypothesis that youth *would not have smoked cigarettes if they had not first used ENDS*.

First, results supporting the gateway hypothesis suffer from strong confounding by one’s pre-existing tendency to use products containing nicotine. How do we know that youth who used ENDS already had a tendency to smoke cigarettes? Research has identified many “risk factors,” or characteristics that increase the chance that a youth will go on to use products containing nicotine. For example, youth who have family members or friends who use these products, youth with certain personality characteristics (e.g. liking to take risks), and youth who experience signs of depression or anxiety are all more likely to use products containing nicotine, including both ENDS and cigarettes [25–27]. There is also evidence of a common genetic predisposition to using both products [10]. All of these would explain why youth who use ENDS also smoke cigarettes.

Therefore, if someone first used ENDS and then started smoking cigarettes, it’s hard to know whether they smoked because *they already had a tendency to smoke* (regardless of whether they used ENDS), or whether using ENDS *caused* them to smoke. The fact that there is a competing explanation to the gateway explanation is described as “confounding.” Researchers must analyze the data very carefully when there is confounding, so they can understand whether ENDS use causes youth to smoke, versus whether the association is *better* described by other risk factors and an overall tendency to use products containing nicotine.

To illustrate the concept of confounding, it may be helpful to discuss a simpler example. Confounding is a common challenge in understanding many different public health questions. There are many examples where one explanation was initially believed, but was then disproven when it was found out that confounding (i.e. an alternative explanation) better explained the results.

For example, one study aimed to examine the potential benefits of certain components of food (“flavonoids”) on brain performance. The study examined how much chocolate (a food high in flavonoids) people consumed on average in each country, and tested whether this was associated with the number of people who won a Nobel Prize (a measure of peak brain performance) in each country [28]. The study reported a strong correlation, where countries with the highest average chocolate consumption had significantly more citizens who won a Nobel Prize. Like the gateway explanation, the idea that chocolate consumption can increase Nobel Prizes had a believable explanation for *how* the effect might work: in this case, through the flavonoids in chocolate improving brain performance.

However, a later study [29] pointed out that this association was confounded by the country’s wealth (as measured by GDP): chocolate and Nobel prizes were associated not because increased chocolate consumption caused more Nobel prizes to be won, but because they are separate (but unrelated) effects of wealth. That is, wealthier countries’ citizens can afford luxury foods like chocolate, and wealthier countries have more funding for education and science. This is referred to as a “spurious correlation;” the follow-up study also reported other spurious correlations, such as between IKEA stores and Nobel Prizes. The latter correlation is more clearly spurious, because there is no believable way that IKEA stores could increase Nobel Prize winning. Yet chocolate consumption is just as incorrect an explanation for Nobel Prize winning as IKEA stores is, *even though the chocolate consumption explanation may sound more believable.*

In a similar vein, an early conceptualizations of the gateway concept – marijuana use leading to subsequent use of “harder” substances – is no longer accepted as a plausible hypothesis [23]. It has since been dismissed for the same reasons: that the association between marijuana use and other substance use reflects common risk factors rather than a causal relationship [23].

### Re-evaluating the evidence for the gateway explanation

The problem of confounding is a severe weakness of the evidence for the gateway explanation, which undermines the strengths of the evidence. In this section, each of the apparent strengths are revisited in light of this weaknesses.

Despite the consistency of findings across studies, each individual study suffers from the same weaknesses discussed above: confounding by risk factors for using products containing nicotine. One study identified 34 risk factors which increase youths’ tendency to use both ENDS and cigarettes, and showed that *none* of the studies supporting a gateway explanation accounted for even a majority of these risk factors [12]. Additionally, no study to date has accounted for the genetic predisposition for using products containing nicotine has been identified [10]. This means that these studies did not fully measure youths’ pre-existing tendency to use products containing nicotine. As a result, the association between ENDS use and cigarette smoking is still confounded; that is, the apparent gateway association might still be better explained by youths’ degree of pre-existing tendency to use products containing nicotine, and not necessarily a causal effect of ENDS use on smoking. The consistency in findings confirms the association between ENDS use and smoking, but does not thereby support the gateway hypothesis’ causal explanation. Consistency across studies is expected – and uninformative – if all studies are biased in the same way.

With respect to some studies’ data having the correct time sequence of ENDS and cigarette use, this is not enough on its own to *prove* that a gateway effect is happening. This is because youth who use ENDS first and then smoke cigarettes probably *already had a strong tendency to use products containing nicotine before ever using ENDS*, because of the many pre-existing risk factors described above.

In fact, there is equally strong evidence for the *reverse* time sequence: youth who smoke cigarettes are significantly more likely to later initiate ENDS use [30, 31]. Similar associations are also present with other products containing nicotine as well (e.g. cigars, smokeless tobacco; [32, 33]), and other substances, e.g. cannabis [34, 35], which is explored in detail below. Taken as a whole, youth who use one product or substance are generally more likely to use another product or substance – regardless of the time sequence – which supports the existence of a general common liability to use substances. In other words, there is nothing unique about the gateway association; it is one of many associations between using one product or substance and using another.

Having a believable explanation as to *how* a gateway effect might work (e.g. through developing nicotine dependence) is important for the overall believability of the gateway explanation, but such an explanation can also make it difficult for researchers to easily recognize when a correlation is spurious (i.e. whether the results are better explained by other factors) – as the case of chocolate consumption and Nobel Prizes showed.

Additionally, the proposed mechanisms (or pathways of action) for how the hypothesized gateway effect might work lack supporting evidence. For example, one study found no evidence that the association between ENDS use and smoking occurs through a pathway of increased nicotine dependence [36]. Similarly, the association was also not significantly related to ENDS renormalizing smoking behavior [37].

Overall, the gateway evidence has an important flaw – that the apparent association between ENDS use and cigarette smoking may be confounded, or better explained by, the degree of youths’ pre-existing tendency to use products containing nicotine. The evidence for the gateway explanation is therefore inconclusive, and other explanations should be considered.

### Common liability explanation

In the section above, the gateway explanation was shown to be inconclusive because of confounding: the evidence *for* gateway may be partially or fully explained by how strongly youths have a pre-existing tendency to use products containing nicotine in general. This leads directly into the common liability explanation: that rather than ENDS use directly *causing* some youth to also smoke cigarettes (as in the gateway explanation), youths’ pre-existing tendency to use products containing nicotine explains why youth who use ENDS also smoke cigarettes. This is referred to as the common liability explanation because the same list of individual characteristics mentioned above are *common* to both ENDS use and cigarette smoking, and having these characteristics is a *liability* (i.e., they are risk factors) for using *any* product that contains nicotine. Therefore, researchers should also consider the common liability explanation when they evaluate the evidence for the gateway explanation.

A higher-level point is worth noting before going into the details of the common liability explanation: unlike the original conceptualization of a gateway effect (i.e., cannabis use to “harder” illicit substances), the current conceptualization involves the *same* substance (i.e., nicotine) in different delivery methods. As such, the gateway hypothesis as applied to ENDS is a fundamentally different concept, asking what could be considered the more trivial (and even tautological) question of whether nicotine administration leads to (a different type of) nicotine administration. From this perspective, the common liability explanation might be considered tautological as well, as there is only one type of “liability,” i.e. for using products that contain nicotine. Nevertheless, since some researchers have argued that the risk factors for the two products do not entirely overlap [38], the common liability framework is relevant for discussing this evidence.

Turning back to the question of gateway vs. common liability explanations, it is difficult to tell conclusively which explanation is true, without being able to know for sure what *would* have happened in a hypothetical world without ENDS. However, there are ways that researchers can analyze the data to make a well-educated guess: this requires taking into account as many common liability factors as possible.

Evidence for the common liability explanation comes from studies that carefully adjust for as many common liability factors as possible, in order to make a well-educated guess for how strong each person’s tendency is to use products that contain nicotine. This will identify which people were probably going to smoke cigarettes anyway, even if they had never used ENDS. If youth who use ENDS are still more likely to smoke cigarettes even after accounting for their pre-existing tendency to use products containing nicotine, this would provide evidence for the gateway explanation. On the other hand, if youth who use ENDS are *no more likely to smoke* after accounting for their degree of pre-existing tendency for using products that contain nicotine, this is evidence for the common liability explanation.

Studies that carefully account for youths’ pre-existing tendency to use products that contain nicotine generally support the common liability explanation. Below are several types of evidence that support the common liability explanation.

### The more common liability factors are accounted for, the weaker the apparent gateway effect becomes

There are at least 34 common liability factors, or characteristics that are associated with both ENDS use and smoking [12]. While gateway studies acknowledge that there is confounding between ENDS use and smoking, and do account for some of these factors, they do not account for all, or even most, of them. A review of gateway evidence found that at most, only approximately one-third of common liability factors were included in most studies (between 5 and 14 of the 34 identified common liability factors) [12]. While sociodemographic factors were almost always included in the analysis, other factors such as mental-health-related symptoms (e.g. internalizing/externalizing disorders) were rarely included [12]. Therefore, it is possible that the common liability factors *not* included in most studies may fully explain the observed association between ENDS use and smoking.

In general, analyses that include more common liability factors find a weaker association between ENDS use and cigarette smoking. For example, Leventhal et al. [39] – the study which adjusted for the most risk factors (14 of the 34 identified in [12]) and still concluded in favor of the gateway explanation – showed that *without* accounting for any common liability factors, the association weakened from an odds ratio of 2.65 (meaning that youth who ever used ENDS have 2.65 times the odds of later smoking than those who never used ENDS), to only 1.75 after adjusting for 14 common liability factors (Table 1). This shows that much of the initial apparent “gateway association” is actually better explained by the pre-existing tendency to use products that contain nicotine, and raises the question of how much further the association would weaken if additional common liability factors were taken into account.

In addition to the number of common liability factors that are adjusted for, a related issue is how accurately those factors are measured. Many common liability factors are not objectively measurable or observable (e.g., how risky one thinks cigarette smoking is), and can only be assessed indirectly via questionnaires. As a result, even if specific questions have been validated, some imperfection remains (e.g., questionnaire responses do not perfectly capture underlying risk perceptions), and this imperfection or “measurement error” can lead to false-positive or spurious associations [40]. Since many common liability factors are questionnaire-based proxy variables, the resulting measurement error may also explain some of what initially appears to be a gateway association.

### Accounting for many common liability factors makes the apparent gateway effect no longer significant

In some studies, including many common liability factors in the analysis weakens the apparent gateway association so much that it is no longer statistically reliable. That is, accounting for youths’ pre-existing tendency to use products that contain nicotine explains their smoking behavior so well that using ENDS does not additionally raise the risk of smoking cigarettes. Table 1 shows some notable examples from the published literature. For example, Sun et al. [13] presented four different analyses of increasing adjustment for common liability variables (for simplicity, only three analyses are presented in Table 1). The minimally-adjusted model (for sociodemographics only) found a positive and significant relationship between e-cigarette use and smoking (AOR = 4.07); however, this association became weaker and ultimately non-significant as more common liability factors were adjusted for. In other words, after using 22 common liability factors to make a statistically-informed estimation of how strong each youth’s tendency to use products that contain nicotine is, using ENDS was *not* significantly associated with any higher chance of later smoking. Similarly, another study found that using a more advanced method of estimating the pre-existing tendency to use products containing nicotine also shows that using ENDS does not pose an additional risk for smoking, beyond that of the pre-existing tendency to smoke [11].

**Table 1:** Impact of adjusting for common liability factors on the apparent gateway association: Examples from published studies

Article and Data	Little/No Adjustment		Intermediate Adjustment		More Thorough Adjustment	
	Details	AOR (95% CI), *p*	Details	AOR (95% CI), *p*	Details	AOR (95% CI), *p*
Sun et al. [13], PATH Waves 4.5-5 *Model*: Lifetime e-cigarette use and P12M smoking	*Adjusted for*: socio-demographics	4.07 (2.86–5.81), *p* < .001	*Adjusted for*: socio-demographics family tobacco use, secondhand smoke, friends’ tobacco use	2.28 (1,60–3.25), *p* < .001	*Adjusted for*: socio-demographics, family tobacco use, secondhand smoke, friends’ tobacco use, cigarette susceptibility, lifetime use of other tobacco products, P12M alcohol and marijuana use	1.35 (0.84–2.16), *p* = .22
Kim & Selya [11], MTF 2015-16 Model: Lifetime e-cigarette use and P30D smoking	Unadjusted association	35.86 (15.85–81.11), *p* < .001	Standard logistic regression, adjusting for: sociodemographics, P30D alcohol and marijuana use, lifetime use of other illicit substances, peer smoking, exposure to health warnings, experienced discipline, risk-seeking behavior, mood, attitutes towards smoking	4.45 (1.73– 11.40), *p* .002	Same set of covariates as in “intermediate adjustment,” but using inverse propensity-weighted regression	2.17 (0.62–7.63), *p* = .228
Leventhal et al. [39], longitudinal survey of California high school students Model: lifetime e-cigarette use and lifetime smoking	Unadjusted association	2.65 (1.73–4.05), *p* < .001	(NA; no intermediate model presented in this article)		Adjusted for: sociodemographics, lifetime substance use, family smoking history, peer smoking, depressive symptoms, impulsivity, delinquent behavior, smoking susceptibility, smoking expectancies, time	1.75 (1.10–2.77), *p* = .02

*Notes* MTF: Montoring the Future. P12M: Past 12 months. P30D: Past 30 days. PATH: Population Assessment of Tobacco and Health. Bold: *p* < .05. Table presents relevant portions of results from each article. The degree of adjustment and number of covariates varies widely across studies; thus, an exact comparison cannot be made across studies, but is grouped here into three broad categories representing increasing adjustment for common liability

These studies provide strong support for the common liability explanation, because they show that youths’ pre-existing tendency to use products containing nicotine *fully explains* their later smoking behavior, and that ENDS use does not make them *more* likely to smoke *over and above that pre-existing tendency*.

### Using products that contain nicotine is associated with using other products that contain nicotine

Studies supporting the gateway explanation focus on the sequence of ENDS use leading to cigarette smoking. However, this is far from the only possible sequence of initiating different products that contain nicotine: it is also possible, for example, for youth to start with cigarette smoking and then start using ENDS (though this would not be considered a gateway sequence). With many other products that contain nicotine also available on the market, such as cigars and smokeless tobacco, there are accordingly many other sequences of initiating use of different products containing nicotine.

Studies that examine the gateway explanation usually do not even look at these other possible sequences of initiating different products that contain nicotine, to see whether starting first with another products that nicotine similarly raises the risk of using a different product containing nicotine. Some studies have, however, more comprehensively studied other possible sequences of initiating different products containing nicotine. These studies generally find similar associations between the use of any two products containing nicotine, *regardless of which product was used first.* For example, a study of youth in the United Kingdom found that those who used ENDS at age 14 were more likely to smoke cigarettes at age 17. However, importantly, the reverse was also true: those who smoked cigarettes at age 14 were more likely to use ENDS at age 17 [30]. ENDS use predicted cigarette smoking just as strongly as the reverse sequence – meaning that regardless of which product was used first, youth who used one product were more likely to use the other product. This is indirect evidence for the common liability explanation, because it shows that using ENDS first (as required in the gateway explanation) is not associated with a unique risk of using other products containing nicotine. Instead, using any product that contains nicotine signals a risk for using another product that contains nicotine products, due to the degree of youths’ general pre-existing tendency to use products containing nicotine.

In fact, the finding that the same people tend to use different products also extends to using substances other than nicotine. For example, using cannabis is a risk for later using products containing nicotine, and vice versa [34, 35, 41]. Since the gateway concept (as originally conceived) involves starting with a more common or licit substance and moving to a “harder” one (e.g. ENDS use to marijuana), the reverse direction (e.g. marijuana to ENDS use) is not interpreted as a gateway effect – yet it is the same type of statistical evidence that is interpreted in favor of a gateway effect of ENDS. In other words, the pathway from ENDS use to cigarette smoking is not unique: it is one of many similar mutual associations between using one product or substance, and using another.

## Other lines of evidence to evaluate the gateway explanation

All of the studies above come from only one type of evidence: studies that ask individual youth whether they used ENDS, and then examine whether they are more likely to smoke cigarettes. However, there are other lines of evidence that can help to tell which explanation is more likely to be correct. It is important to “triangulate” [15, 16] across these different ways of looking at the gateway question, to see whether they support or contradict the gateway explanation. This section considers other lines of evidence.

### Population trends in smoking prevalence

If the gateway explanation were true, it would mean that as ENDS were introduced and came to be used by youth, one should see a rise in youth smoking compared to what would have occurred without ENDS – because using ENDS is hypothesized to *cause* some of them to smoke, when they would not otherwise have smoked. This prediction can be tested by looking at how common cigarette smoking is in the youth population (“smoking prevalence”), and how that has changed over time (“population-level trends”).

Although it is not possible to know for certain what the youth smoking prevalence would be in an alternative world without ENDS, there are ways of making statistically-informed estimates. For example, researchers can look at the smoking prevalence trends before ENDS appeared, and forecast what those trends would have looked like now without the potential changes caused by ENDS use. These forecasts can then be compared with actual smoking prevalence trends: if the gateway explanation were correct, then the actual smoking prevalence would be higher than the forecasted, or expected, smoking prevalence in a world without ENDS.

However, studies consistently find the *opposite* – that youth smoking prevalence is *lower* now than we would have expected from projecting the trends before ENDS became available [42–44] as summarized in Table 2. For example, smoking prevalence trends were already declining prior to ENDS entering the market, but Levy and colleagues [14] showed that current smoking prevalence declined two to four times faster after ENDS use became common. This evidence is consistent with the idea that using ENDS may have displaced cigarette smoking among youth and *diverted* them away from ever smoking cigarettes (“diversion effect”).

**Table 2:** Examples of published studies showing more rapid declines in youth smoking coincident with the introduction of ENDS

Article	Location, Data	Pre-trend in cigarette smoking		Post-trend in cigarette smoking		Comments
		Year(s)	Annual Change	Year(s)	Annual Change	
Levy et al. [14]	USA, MTF 12th grade	2004–2013	-4.6%	2014–2017	-14.1%	“Thus, the downward trend was more than three times greater in the vaping period” (14.1%) than the long-term trend (4.6%).
	USA, MTF 10th grade	2004–2013	-5.8%	2014–2017	-19.8%	“…more than three times the relative reduction in the vaping period (19.8%=5.8%+14.0%) than the preceding period (5.8%)”
	USA, NYTS high school	2000–2013	-8.2%	2014–2017	By -22.0%	“With the vaping period modelled as a simple reduction in smoking for the YRBS and NYTS analyses, we obtained a 52% lower past 30-day smoking rates in the vaping period for YRBS and a 22% reduction for NYTS”
	USA, YRBS high school	2003–2017	-6.2%	2014–2017	By **-**52.0%	
Foxon & Selya [42]	USA, NYTS	1999–2009	Projected smoking prevalence in 2018: ~4.25%	2010–2018	Actual smoking prevalence in 2018: ~1.9%	Analyses compared actual data vs. counterfactual projections (based on pre-period trends) using exponential trend modeling.
Meza et al. [43]	USA, MTF 12th grade boys	2006–2012	-1.6%	2012–2019	-17.4%	
	USA, MTF 10th grade boys	2005–2011	-0.7%	2011–2019	-17.9%	
	USA, MTF 8th grade boys	1996–2011	-8.8%	2011–2019	-17.3%	
	USA, MTF 12th grade girls	1997–2012	-7.2%	2012–2019	-17.5%	
	USA, MTF 10th grade girls	1997–2012	-9.5%	2012–2019	-16.3%	
	USA, MTF 8th grade girls	1991–1996	10.9%	1996–2019	-10.8%	
Wagner & Clifton [44]	USA, American Lung association Tobacco Trend Brief	1990–2009	Projected smoking prevalence in 2019: ~12%	2010–2019	Actual smoking prevalence in 2019: ~6%	Analyses compared actual data vs. counterfactual projections (based on pre-period trends) using dynamical systems modeling.

*Notes* MTF: Montoring the Future. NYTS: National Youth Tobacco Survey. YRBS: Youth Risk Behavior Survey

Another study [45] used simulation modeling to test different scenarios of how youth ENDS use could affect established smoking prevalence, including a gateway scenario that increased smoking in proportion to ENDS use, and a diversion scenario that prevented smoking in proportion to ENDS use. When each of these scenarios was compared to real data on youth smoking, the gateway scenario predicted far higher prevalence of youth smoking than was actually the case. In fact, a rather large diversion scenario was necessary in order to explain actual data, with an exponential time constant of 55% (meaning that approximately 55% of youth who use ENDS are diverted from ever accumulating an established smoking history within the next 3 years). It is also possible to have gateway and diversion effects at the same time, though the diversion effect must necessarily be even stronger (exponential time constant of 65%) in order to match *actual* smoking trends.

A number of other studies using different analytical methods and different datasets have also found that youth smoking has declined more in recent years after ENDS use became common [46–48]. It is difficult to distinguish between competing causes of this more rapid decline, which could include tobacco control policies. For example, in the US, the Family Smoking Prevention and Tobacco Control Act (FSPTCA) in 2009 could have contributed to the decline in youth smoking, but does not seem to explain the full magnitude of the decline: estimates of FSPTCA’s effect on youth cigarette smoking are smaller (incremental decline of 5.2% per year^1^ [43], versus ~ 10% [43]^2^). Moreover, the finding that more rapid declines in youth cigarette smoking coincided with the introduction of ENDS is remarkably consistent across countries with different tobacco regulations [50–52], suggesting that tobacco policies cannot fully account for the more rapid recent declines. Regardless of the cause of these recent accelerated declines in youth smoking, the consistency of these recent declines across settings is at odds with a substantial gateway effect that is detectable at the population level.

### Absence of evidence vs. evidence of absence

While the above evidence does not *disprove* the gateway explanation (it only fails to support it), other studies do provide evidence *against* the gateway explanation. Beard et al. [53] found no population-level increase in smoking after youth began taking up ENDS, and performed a follow-up analysis that indicated the non-significant finding was likely due to there being no true gateway effect (as opposed to the possibility that there is a true effect, but the analysis being unable to detect it). While this analysis could not rule out a very small gateway effect, it nevertheless provides strong evidence *against* a substantial gateway effect.

### If there is a gateway effect, what is its population-level impact?

As described above, the gateway explanation is that using ENDS causes some youth to smoke. But if this were occurring, how much of an impact would it be having at the population level? There are multiple factors determining how well, or how poorly, the results of the above analyses translate into real-world effects in the population. One factor is how *generalizable* the participants analyzed in research studies are – i.e., how well they represent the entire population of youth in the real world. Many research studies are assumed, or presented, as having high generalizability to all youth, by virtue of using nationally-representative survey data (e.g. PATH); however, Dautzenberg et al. recently pointed out that the majority of data are (appropriately) discarded in longitudinal analyses of the gateway association [54]. Specifically, in order to ensure the right time sequence (e-cigarette use at the earlier timepoint (“T1”) and cigarette smoking being initiated some time later (by “T2”)), these analyses must exclude youth who were already using *both* products before T1 (and whose time sequence was unknown) – which excluded 64.3% of youth who used ENDS at T1. Additionally, most youth who started smoking between T1 and T2 had never used ENDS (74.1%), meaning that they started smoking for reasons other than a gateway from ENDS. After accounting for all data exclusions, the hypothesized gateway effect could explain, at most, only 5.3% of smoking at T2 – demonstrating that even if the gateway effect were occurring, it could only explain a small fraction of overall youth smoking. Moreover, Dautzenberg et al. re-analyzed data without these data exclusions, and came to the opposite conclusion: “When the whole original cohort is considered, the *Dispersion effect* is dominant and totally masks a possible *Gateway effect*” [54].

A second factor in examining the real-world population impact of the hypothesized gateway effect is what level of smoking “counts” – one puff of a cigarette? A whole cigarette? Smoking at all in the past year or past month? Smoking frequently (20 + days out of the past 30 days)? Established smoking (100 + cigarettes/lifetime)?

From a public health perspective, it is prolonged smoking that is the main concern, because that has serious health risks. For example, one study reported that adults who quit smoking by age 35 avoided the vast majority of premature mortality attributed to cigarette smoking [55]. Much lower cutoffs are used for youth, since they have not yet had time to develop such extensive smoking history, and experimentation *could* lead to regular use.

However, it is a matter of debate which cutoffs are best to use when studying youth smoking: a more lenient criteria for smoking (e.g. past-year smoking) will affect more of the population, and seemingly affect more youth in the population. The gateway literature predominantly focuses on lenient measures of smoking: for example, NASEM reviewed 10 gateway studies in 2018; 8 of these used *ever*-smoking (a puff or a whole cigarette) – the lowest possible cutoff – as the outcome, while 2 used past-30-day smoking. However, the majority of youth who ever *try* a cigarette are merely experimenting: for example, one analysis showed that approximately 2/3 of youth who ever tried did *not* go on to regular or established use [56]. Thus, even if a small gateway effect impacts a minority of youth, this effect mainly relates to experimentation, and most of those youth will not go on to smoke regularly.

Indeed, studies that use more stringent definitions of smoking less often find an apparent gateway association. For example, Kim and Selya [11], after adjusting for the pre-existing tendency to use products containing nicotine, found a remaining (though not necessarily causal) association between ENDS use and *ever*-smoking, whereas common liability factors *fully* explained the association with the more stringent measure of past-30-day smoking. Very few studies examined potential gateway effects on more stringent measures of smoking such as established use. However, vanishingly few youth met more stringent definitions of smoking: for example, one study [33] found that only approximately 0.2% of youth met the most stringent criteria (smoking cigarettes at two separate follow-ups, smoking > 100 cigarettes/life, and smoking on 20 + days out of the past 30 days) – underscoring that if there were a gateway effect for some youth, it would affect a very small percentage of youth.

Overall, it is important to choose an appropriate smoking cutoff with which to examine a gateway effect. Gateway studies should choose more stringent measures of smoking that “weed out” experimental use and better capture more problematic smoking behavior.

## Conclusions

A major concern about ENDS use is the hypothesis that they may act as a gateway to cigarette smoking among youth. Evidence supporting this gateway explanation is based on findings that youth who use ENDS are also more likely to smoke cigarettes. However, this evidence suffers from an important flaw: these studies fail to fully account for some youths’ pre-existing tendency to use nicotine products – either by not including sufficient common liability variables, or not accurately *measuring* common liability – and inappropriately interpret the results as ENDS use *causing* some youth to smoke. The common liability explanation is the primary alternative way of interpreting this evidence. In support of the common liability explanation, the more thoroughly researchers account for youths’ pre-existing tendency to use products that contain nicotine, the weaker the potential unique effect of ENDS use becomes – to the point where, in some studies, ENDS use poses *no additional risks*. Different lines of evidence also contradict the gateway explanation and support the common liability explanation, namely population-trend modeling studies that show smoking to be less common among youth now, than would be expected if ENDS had never become available. In fact, this raises the question about whether ENDS are *diverting* youth away from ever smoking cigarettes, which is an important area for future research. Nevertheless, the evidence overall cannot rule out a small gateway effect, but if it exists, it is small and limited in scope, and, when considering ENDS’ overall population impact, this possible small detrimental effect would likely be outweighed by ENDS’ beneficial effects on helping adults who smoke switch away from cigarettes [57, 58]. In any case, common liability factors explain at least a substantial proportion of the association between ENDS use and cigarette smoking. As a result, and given that cigarettes and ENDS are substitutes, policies that are based on misinterpreting this association as a causal gateway effect are likely to be ineffective and even unintentionally harmful, in terms of increased *cigarette* smoking rates.

## Data Availability

No datasets were generated or analysed during the current study.

## Footnotes

1 Calculated from Rossheim et al. [49] as the incremental decline of 43% decline over 8.25, or 5.2% per year.

2 Calculated from Meza et al. [43] as the difference between slopes before vs. after the closest joinpoint: 8th graders: decline increase from 6.1% in 2002–2011 to 14.2% in 2011–2019, or an incremental decline of 7.1%; 10th graders: decline increased from 3.1% in 2004–2011 to 14.9% in 2011–2019, or an incremental decline of 11.8%; 12th graders: decline increased from 4.0% in 2003–2013 to 15.0% in 2013–2019, or an incremental decline of 11.0%.

## Abbreviations

ENDS: Electronic Nicotine Delivery Systems
FDA: U.S. Food and Drug Administration
FSPTCA: Family Smoking Prevention and Tobacco Control Act
MTF: Monitoring the Future
NASEM: National Academies of Sciences, Engineering, and Medicine
NYTS: National Youth Tobacco Survey
PATH: Population Assessment of Tobacco and Health Study
YRBS: Youth Risk Behavior Survey
